# Deregulation of manganese superoxide dismutase (SOD2) expression and lymph node metastasis in tongue squamous cell carcinoma

**DOI:** 10.1186/1471-2407-10-365

**Published:** 2010-07-09

**Authors:** Xiqiang Liu, Anxun Wang, Lorenzo Lo Muzio, Antonia Kolokythas, Shihu Sheng, Corrado Rubini, Hui Ye, Fei Shi, Tianwei Yu, David L Crowe, Xiaofeng Zhou

**Affiliations:** 1 Center for Molecular Biology of Oral Diseases, College of Dentistry, University of Illinois at Chicago, Chicago, IL, USA; 2 Research Institute & the Affiliated Hospital of Stomatology, Sun Yat-Sen University, Guangzhou, China; 3 Department of Oral and Maxillofacial Surgery, the First Affiliated Hospital, Sun Yat-Sen University, Guangzhou, China; 4 Department of Surgical Sciences, Faculty of Medicine, School of Dentistry, University of Foggia, Foggia, Italy; 5 Department of Oral and Maxillofacial Surgery, College of Dentistry, University of Illinois at Chicago, Chicago, IL, USA; 6 Department of Pathology, Faculty of Medicine, Marche Polytechnic University, Ancona, Italy; 7 Division of Epidemiology and Biostatistics, School of Public Health, University of Illinois at Chicago, Chicago, IL, USA; 8 Department of Biostatistics and Bioinformatics, Rollins School of Public Health, Emory University, Atlanta, GA, USA; 9 Graduate College, UIC Cancer Center, University of Illinois at Chicago, Chicago, IL, USA

## Abstract

**Background:**

Lymph node metastasis is a critical event in the progression of tongue squamous cell carcinoma (TSCC). The identification of biomarkers associated with the metastatic process would provide critical prognostic information to facilitate clinical decision making. Previous studies showed that deregulation of manganese superoxide dismutase (SOD2) expression is a frequent event in TSCC and may be associated with enhanced cell invasion. The purpose of this study is to further evaluate whether the expression level of SOD2 is correlated with the metastatic status in TSCC patients.

**Methods:**

We first examined the SOD2 expression at mRNA level on 53 TSCC and 22 normal control samples based on pooled-analysis of existing microarray datasets. To confirm our observations, we examined the expression of SOD2 at protein level on an additional TSCC patient cohort (n = 100), as well as 31 premalignant dysplasias, 15 normal tongue mucosa, and 32 lymph node metastatic diseases by immunohistochemistry (IHC).

**Results:**

The SOD2 mRNA level in primary TSCC tissue is reversely correlated with lymph node metastasis in the first TSCC patient cohort. The SOD2 protein level in primary TSCC tissue is also reversely correlated with lymph node metastasis in the second TSCC patient cohort. Deregulation of SOD2 expression is a common event in TSCC and appears to be associated with disease progression. Statistical analysis revealed that the reduced SOD2 expression in primary tumor tissue is associated with lymph node metastasis in both TSCC patient cohorts examined.

**Conclusions:**

Our study suggested that the deregulation of SOD2 in TSCC has potential predictive values for lymph node metastasis, and may serve as a therapeutic target for patients at risk of metastasis.

## Background

Oral squamous cell carcinoma (OSCC) is a complex disease arising in various sites. Tumors from these different sites have distinct clinical presentations and outcomes, and are associated with different genetic characteristics [1]. In this study, we focused on tongue SCC (TSCC), one of the most common sites for OSCCs. The TSCC is significantly more aggressive than other forms of OSCCs, with a propensity for rapid local invasion and spread [2].

Lymph node metastasis is identified as the single most adverse independent prognostic factor in patients with OSCC [3]. Currently, the detection of nodal metastasis is based on clinical and radiographic examination as well as histopathologic examination of the surgical specimen if a neck dissection is performed. However, regional lymph node metastasis cannot always be reliably detected by these routine examinations. This points to the immediate need for new diagnostic strategies to better identify those tumors with potentially higher metastatic abilities. Since several genes have been reported in retrospective trials to yield prognostic information independently of the TNM classification, it is reasonable to hypothesize that molecular "fingerprints" could exist that might define sub-groups of patients with significantly more aggressive disease.

The effects of redox state play important roles in malignancies. Superoxide dismutase 2 (SOD2) has been considered as one of the most important antioxidant enzymes that regulate the cellular redox state in normal and tumorigenic conditions. Studies suggested that alteration in SOD2 level may influence the metastatic potential of tumor cells via activating mitogen-activated protein kinases (MAPK), and regulating the expression of matrix metalloproteinase (MMP) gene family members (including MMP-1 and MMP-9) [4-7]. The object of this study is to evaluate whether the expression level of SOD2 is correlated with metastatic status in TSCC patients. We first performed pooled-analysis on existing TSCC microarray datasets to explore the potential associations between of SOD2 mRNA levels and clinicopathological characteristics. We then tested an additional independent TSCC patient group to confirm and further elucidate the role of SOD2 in TSCC.

## Methods

### Patients

Two TSCC patient groups (total n = 153), 31 cases of dysplasia of the tongue and 15 normal tongue biopsies were utilized in this study. Clinical characterization of the patients is summarized in Table 1. The cohort #1 was used for evaluating the SOD2 expression at mRNA level. This group consists of 53 TSCC cases and 22 matching normal tissues where the microarray data were either generated from our previous study [8] or downloaded from GEO database [9-11]. The cohort #2 was used for assessing SOD2 expression at protein level using immunohistochemical assay. This group includes 100 TSCC patients who underwent surgery at the First Affiliated Hospital, Sun Yat-Sen University (Guangzhou, China) between 1998 and 2006. The tumor extent was classified according to the TNM system by UICC, and the tumor grade was classified according to the WHO classification of histological differentiation. Among this 100 TSCC cases, forty cases were diagnosed with lymph node metastasis (pN+). Of the 40 pN+ cases, thirty two cases have available biopsies of the lymph node metastasized diseases for the immunohistochemical assay. In addition, 31 cases of dysplasia of the tongue and 15 normal tongue biopsies were also obtained from the same clinic for the immunohistochemical assay. This study was approved by the ethical committee at Sun Yat-Sen University, and the Institutional Review Boards (IRB) at University of Illinois at Chicago.

**Table 1:** Clinical Characterization of the cohorts^†^

		Cohort #1		Cohort #2		
		**TSCC**	**Normal**	**TSCC**	**Premalignant**	**Normal**

**Age**	Median (range)	57 (32-82)	56 (37-78)	53 (21-77)	51 (30-82)	47 (30-72)

**Gender**	Male: n (%)	39 (73.58)	13 (59.09)	60 (60.00)	17 (54.84)	6 (40.00)
	Female: n (%)	14 (26.42)	9 (40.91)	40 (40.00)	14 (45.16)	9 (60.00)

**T stage**	Stage 4: n (%)	30 (56.60)		6 (6.00)		
	Stage 3: n (%)	4 (7.55)		15 (15.00)		
	Stage 2: n (%)	12 (22.64)		51 (51.00)		
	Stage 1: n (%)	7 (13.21)		28 (28.00)		

**N stage **(Pathological)	Stage 2: n (%)	23 (43.40)		18 (18.00)		
	Stage 1: n (%)	4 (7.55)		22 (22.00)		
	Stage 0: n (%)	26 (49.05)		60 (60.00)		

**Clinical stage**	Stage 4: n (%)	39 (73.58)		21 (21.00)		
	Stage 3: n (%)	4 (7.55)		27 (27.00)		
	Stage 2: n (%)	7 (13.21)		26 (26.00)		
	Stage 1: n (%)	3 (5.66)		26 (26.00)		

**Grade^‡^**	Well: n (%)	NA		51 (51.00)		
	Mod: n (%)	NA		30 (30.00)		
	Poor: n (%)	NA		19 (19.00)		

^† ^The M stage data for TSCC patients is not available.

^‡ ^The Grade data is not available for TSCC cohort #1.

### Pooled-analysis to extract SOD2 expression values from existing microarray datasets

The CEL files from all datasets (53 TSCCs and 22 normal tongue samples) were imported into the statistical software R 2.4.1 [12] using Bioconductor [13]. The pooled-analysis was performed as described [14], and the genome-wide expression pattern has been presented in our previous study [8]. In brief, the Robust Multi-Array Average (RMA) expression measures [15] were computed after background correction and quantile normalization for each microarray dataset. Then, expression values of the overlapping probesets between U133A and U133 Plus 2.0 arrays were extracted. Probeset-level quantile normalization was performed across all samples to make the effect sizes similar among the four datasets [14]. The expression values for probesets corresponding to SOD2 gene (215223_s_at and 216841_s_at) and for Ki67 gene (212020_s_at, 212021_s_at, 212022_s_at, and 212023_s_at) were then extracted from each datasets. Relative expression level for SOD2 and Ki67 genes for each TSCC samples were computed as follows:

First, for every probeset, we found the mean expression level in normal samples, , where *j *denotes the probeset. Second, the expression values derived from the probeset were normalized by the mean normal expression level,, where *i *denotes the TSCC sample and *j *denotes the probeset. Third, for every TSCC sample, the expression value of the gene was assigned by taking the average over the normalized expression levels of the probesets representing the same gene, , where n is the number of probesets for each gene (*n = 2 *for SOD2 and *n = 4 *for Ki67).

### Immunohistochemical analysis

Tissue samples were dehydrated in an ethanol series, cleared in xylene, and embedded in paraffin. Five-micrometer sections were prepared and mounted on poly-L-lysine-coated slides. Representative sections were stained with H&E and histologically evaluated by a pathologist. Immunohistochemical analysis was done using a commercially available kit (Invitrogen, Carlsbad, CA). Sections were incubated at 60°C for 30 min and deparaffinized in xylene. Endogenous peroxidase activity was quenched by incubation in a 9:1 methanol/30% hydrogen peroxide solution for 10 min at room temperature. Sections were rehydrated in PBS (pH 7.4) for 10 min at room temperature. Sections were blocked with 10% normal serum for 10 min at room temperature followed by incubation with anti-SOD2 and anti-Ki67 antibodies (ABCam) at a dilution of 1:200 for 16 h at room temperature. After washing thrice in PBS, the sections were incubated with secondary antibody conjugated to biotin for 10 min at room temperature. After additional washing in PBS, the sections were incubated with streptavidin-conjugated horseradish peroxidase enzyme for 10 min at room temperature. Following final washes in PBS, antigen-antibody complexes were detected by incubation with a horseradish peroxidase substrate solution containing 3,3'-diaminobenzidine tetrahydrochloride chromogen reagent. Slides were rinsed in distilled water, coverslipped using aqueous mounting medium, and allowed to dry at room temperature. The relative intensities of the completed immunohistochemical reactions were evaluated using light microscopy by 3 independent trained observers who were unaware of the clinical data. All areas of tumor cells within each section were analyzed. All tumor cells in ten random high power fields were counted. A scale of 0 to 3 was used to score relative intensity, with 0 corresponding to no detectable immunoreactivity and 1, 2, and 3 equivalent to low, moderate, and high staining, respectively. As for Ki67, nuclear staining was evaluated by calculating the percentage of positive nuclei (Ki67 index).

### Statistical analysis

Spearman Correlation Coefficient was used to assess correlations among the gene expression and clinical and histopathological parameters. One-way ANOVA was used to assess the association of SOD2 expression with clinical and histopathological parameters (e.g., TNM and grade). The normality of the observed SOD2 expression data were tested by quantile-quantile plot (QQ plot) (Additional File 1).

## Results

### The SOD2 mRNA level in TSCC

**Figure 1:**
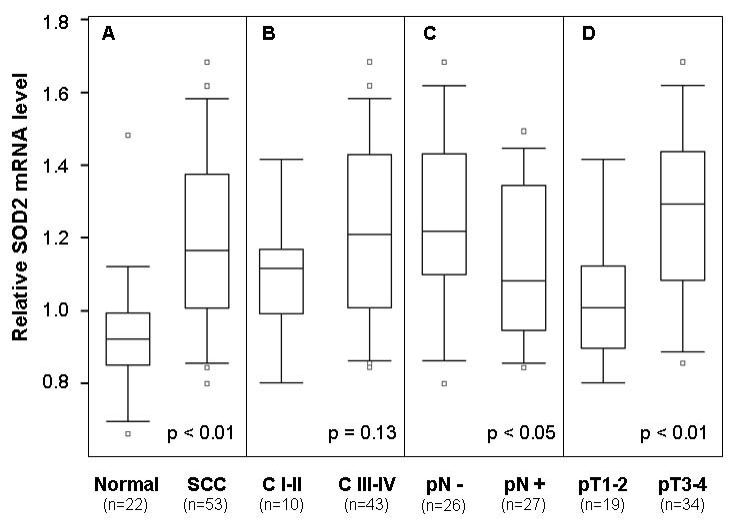
**mRNA expression of SOD2 in TSCC based on pooled-analysis**. Pooled-analysis was performed to extract the relative expression level of SOD2 mRNA from microarray datasets of 53 TSCCs and 22 normal control samples as described. Box plots were presented for comparing the SOD2 mRNA levels of SCC and normal control samples (**A**), clinical stage I-II and clinical stage III-IV SCC samples (**B**), SCC samples with (pN+) and without lymph node metastasis (pN-) (**C**), and SCC samples with different pT stages (**D**). The p-values were computed using one-way ANOVA. The boxes represent 25^th ^to 75^th ^percentile of the observations, and the lines in the middle of the box represent the median. The whiskers represent maximum (or minimum) observations below (or above) the 1.5 times of the interquartile range (IQR), respectively. Outliers are also indicated in the plots as small circles.

Pooled-analysis was performed on previous existing microarray datasets to determine the SOD2 mRNA levels in TSCC (n = 53) and normal tongue samples (n = 22). As illustrated in Figure 1A, the SOD2 expression is significantly higher in TSCCs than in normal tissue samples. The expression level of SOD2 is somewhat higher in late stage (stage III and IV) than early stage disease (stage I and II), but the difference is not statistically significant (Figure 1B). Statistical significant reduction in SOD2 mRNA level was observed in the primary disease tissues with positive node metastasis status (pN+) as compared to those with negative status (pN-) (Figure 1C). Increased SOD2 expression was also observed in larger (T3-4) vs. smaller (T1-2) primary tumors (Figure 1D).

### The SOD2 protein level in TSCC

**Figure 2:**
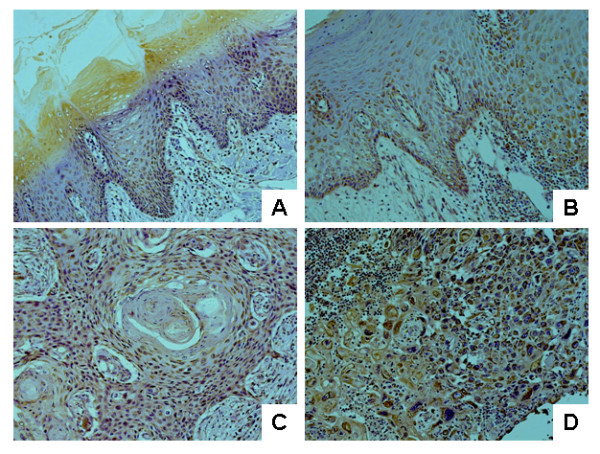
**OD2 expression in normal tongue, premalignant dysplasia, primary TSCC and lymph node metastasis tissue samples**. Immunohistochemical analysis for SOD2 was performed as described in the Material and Methods on normal tongue mucosa (**A**), premalignant dyslasia (**B**), primary TSCC, and (**C**) lymph node metastasis tissue samples (**D**). Representative Images (×200) were shown.

To confirm our observations and further elucidate the role of SOD2, the expression of the SOD2 gene was examined by immunohistochemistry (IHC) in an additional TSCC patient cohort (total n = 100), as well as 31 premalignant dysplasias, 15 normal tongue mucosa, and 32 cases of lymph node metastatic disease (Table 2). As illustrated in Figure 2A, SOD2 IHC staining was sparsely observed in the cytoplasm of epithelial layers with the exception of the superficial layers for the normal tongue tissue. Among 15 normal tongue mucosa samples that we examined, 9 cases (60%) exhibited weak SOD2 staining, 5 cases (33.3%) exhibited moderate staining, and 1 case (6.7%) showed no staining. All premalignant dysplasia cases showed positive IHC staining in all layers of the mucosa (Figure 2B). The percentage of positive cells and the staining intensity were increased as compared to normal mucosa. Of the 31 premalignant cases, 3 cases (9.7%) showed strong SOD2 staining, 14 cases (45.1%) showed moderate staining, 11 cases (35.5%) showed weak staining and 3 cases (9.7%) showed no staining. Among TSCC primary cases, the intensity of SOD2 staining varied dramatically. Of the 100 TSCC samples evaluated, 25 cases (25%) showed strong SOD2 staining, in 29 cases (29%) showed moderate staining, 42 cases (42%) showed weak staining and 4 cases (4%) showed no staining. Predominantly cytoplasmic staining for SOD2 was observed in the less-differentiated cells located at the periphery of carcinomatous clusters but negative staining was observed for areas of keratin pearls (Figure 2C). For lymph node metastatic disease, homogeneous staining involved almost all the metastatic tumor cells in the affected lymph nodes (Figure 2D). Among 32 lymph node metastases that we examined, strong staining was observed in 10 cases (31.25%), moderate staining in 11 cases (34.4%), weak staining in 10 cases (31.25%), and negative staining in 1 case (3.1%).

**Table 2:** The protein expression of SOD2 in TSCC^†^

	Strong	Moderate	Weak	No
**Normal: **n (%)	0 (0.00)	5 (33.33)	9 (60.00)	1 (6.67)
**Premalignant: **n (%)	3 (9.68)	14 (45.16)	11 (35.48)	3 (9.68)
**TSCC: **n (%)	25 (25.00)	29 (29.00)	42 (42.00)	4 (4.00)
**LN metastasis: **n (%)	10 (31.25)	11 (34.38)	10 (31.25)	1 (3.12)

^† ^The SOD2 levels were measured by immunohistochemistry (IHC) on cohort #2. The cases were categorized based on relative SOD2 level (IHC staining) as strong, moderate, weak, and no staining.

### Correlation between SOD2 expression and clinicopathological characteristics in TSCC

Correlations were tested among gene expression (e.g., SOD2 and Ki67), clinical and pathological features for both TSCC patient groups (Table 3). For patient group #1 (n = 53), the SOD2 mRNA level was positively correlated with the T stage and inversely correlation with pN. For patient group #2 (n = 100), the SOD2 protein level was inversely correlated with pN, clinical stage, differentiation (grade) and Ki67 index. It is important to notice that of the 2 patient groups examined (total n = 153), inverse correlation was consistently observed between SOD2 expression and pN. As illustrated in Table 4, statistical analysis revealed that the SOD2 expression level in primary TSCC is associated with pN status in both patient groups (p = 0.043 and p = 0.0004, respectively).

**Table 3:** Correlations among clinical features and SOD2 gene expression of primary TSCC^†^

Age	Gender	pT stage	pN stage	C stage	Grade	Ki67^‡^	SOD2^§^
Patient cohort # 1 (n = 53)							
**Age**	0.06	-0.07	0.08	0.03	na	0.16	0.14
**Gender**		0.09	-0.25	-0.07	na	0.01	0.20
**pT stage**			0.05	0.65 *	na	-0.18	0.49 *
**pN stage**				0.49 *	na	0.21	-0.28 *
**C stage**					na	-0.09	0.19
**Grade**						na	na
**Ki67**							-0.01
**SOD2**							
							
Patient cohort #2 (n = 100)							
**Age**	0.12	-0.05	-0.10	-0.01	-0.01	-0.01	-0.16
**Gender**		0.11	0.04	0.11	0.07	-0.05	-0.02
**pT stage**			0.42 *	0.77 *	-0.16	-0.01	0.04
**pN stage**				0.80 *	-0.05	0.09	-0.35 *
**C stage**					-0.07	0.05	-0.21 *
**Grade**						0.05	-0.30 *
**Ki67**							-0.28 *
**SOD2**							

**^† ^**Spearman Correlation Coefficients.

^‡ ^The Ki67 index for Patient Cohort #1 was based on the relative mRNA level. The Ki67 index for Patient Cohort #2 was based on Immunohistochemical analysis.

^§ ^The expression levels of SOD2 for Patient Cohort #1 were based on the relative mRNA level. The expression levels of SOD2 for Patient Cohort #2 were generated from Immunohistochemical analysis.

* p < 0.05. The p values were computed using Fisher's transformed z-score test.

na: data not available.

**Table 4:** Association of reduced SOD2 expression in primary TSCC and lymph node metastasis (pN)^†^

	n	SOD2^‡^	
		
		Average	Variance
Patient Cohort #1			
**pN +**	27	1.18	0.19
**pN -**	26	1.29	0.21
			(p = 0.043)
			
Patient Cohort #2			
**pN +**	40	1.06	0.57
**pN -**	60	1.62	0.58
			(p = 0.0004)

^† ^One-way ANOVA was used to assess the association of SOD2 with pN.

^‡ ^The expression levels of SOD2 for Patient Cohort #1 were based on the relative mRNA level. The expression levels of SOD2 for Patient Cohort #2 were generated from Immunohistochemistry. The p-values were computed using one-way ANOVA.

## Discussion and Conclusions

An essential characteristic of cancer is the ability to invade surrounding tissues and metastasize to regional lymph nodes and distant sites [16]. Detection of local lymph node metastasis is pivotal for choosing appropriate treatment, including individuals diagnosed with OSCC. It is believed that the underlying molecular features of the tumors play an essential role in determining the aggressiveness of the tumors. Previous studies suggested that the deregulation of SOD2 gene expression is a common event in OSCC and appears to be associated with invasion [8,17,18].

In our present study, by focusing on cancer from just one anatomic site (tongue), we were able to partially minimize the genetic variation and microenvironment effects among intraoral sites. We made two important observations. First, deregulation of SOD2 gene expression is a progressive event in the tumorigenesis of TSCC. Our study confirmed that the SOD2 level is increased in TSCC when compared to normal tissue. The SOD2 level is further enhanced in metastatic diseases when compared with primary tumors. Second, the association of reduced SOD2 expression in primary TSCC and the presence of lymph node metastasis was consistently observed in both patient groups. Similar observations have been made in esophageal cancer and melanoma, where inverse correlations were observed between SOD2 expression and metastatic potential of the cancer cells [19,20]. Nevertheless, our sample size (total n = 153) is relatively small. Further validation studies with larger sample size and additional stratification to control potential confounding factors (e.g., age, gender, ethnicity, smoking histories) are needed to validate our findings.

Accumulating evidence indicates that the deregulations of the SOD2 expression and the intracellular redox state play important roles in the progression of TSCC. Our previous Gene Ontology-based analysis suggested that redox related biological activities, such as superoxide release (GO:0042554), hydrogen peroxide metabolism (GO:0042743), and response to hydrogen peroxide (GO:0042542), are significantly altered in TSCC [8]. However, the precise roles of SOD2 and redox state in malignancies are not fully explored. In theory, reducing the oxidative stress may prevent DNA degeneration and therefore prevent the development of cancer. However, doing so may also offer increased growth potential to tumor cells and protect them from an excess of reactive oxygen species (ROS), which would otherwise lead to apoptosis or necrosis. SOD2 is mainly present in the mitochondrial matrix, where most oxygen is consumed and oxidative stress is most evident. The role of SOD2 in carcinogenesis has been widely studied but remains ambiguous. While some in vitro studies have reported a protective role of SOD2 against tumor progression in several type of cancer cell lines [21-26], in vivo studies suggest more complicated roles of SOD2 in tumorigenesis. Increased SOD2 levels have been observed in gastric, brain astrocytic, and colorectal carcinomas, and is often associated with metastasis and poor prognosis [27-35]. The status of SOD2 levels in breast cancer is not clear, with some studies showing increased SOD2 [36], while others showing a decrease in SOD2 [37]. There seems to be a reduction in SOD2 levels also in prostatic carcinomas when compared to healthy tissue [38,39]. It has been suggested that SOD2 may influence the metastatic potential of tumor cells via regulating the expression of MMP gene family members (including MMP-1 and MMP-9) [5-7]. Interestingly, a single nucleotide polymorphism (SNP) that creates an Ets site at the promoter region of the MMP-1 gene has been shown to be responsible for the SOD2 dependent MMP-1 expression [6]. This SNP has been extensively investigated and has been shown in several populations to be associated with OSCC susceptibility and aggressiveness [40-43]. Studies also showed that hydrogen peroxide generated by SOD2 can activate MAPK and transcriptional factors such as c-fos, c-jun and NFκB [4,44,45]. Further studies are needed to fully explore the functional roles of these molecular regulators in the metastasis of TSCC.

In summary, our study confirmed that the SOD2 level is increased in TSCC when compared to normal tissue. The SOD2 level is further enhanced in metastatic disease when compared with primary tumors. In primary TSCC, reduced SOD2 expression is associated with presence of lymph node metastasis. Taken together, our findings suggested that SOD2 may be useful as a biomarker for the molecular classification of TSCC. Nevertheless, further validation studies with larger, independent sample sets and additional stratification to control potential confounding factors are needed to validate our findings.

## Competing interests

The authors declare that they have no competing interests.

## Authors' contributions

AW, HY, LLM and XZ conceived the idea for the project and drafted the manuscript. AW, XL, SS, CR, AK, HY and TP performed the laboratory analyses. FS, TY and XZ conducted statistical analyses. AW, XL, LLM, AK and DC provided discussions on clinical relevance. AW, XL, DC and XZ drafted the manuscript. All authors read and approved the final manuscript.

## Pre-publication history

The pre-publication history for this paper can be accessed here:

http://www.biomedcentral.com/1471-2407/10/365/prepub

## Supplementary Material

Additional file 1 **Quantile-quantile plot (Q-Q plot) for the normality testing on the SOD2 expression values from cohort #1 and cohort #2**. The normal Q-Q plots were constructed to compare standardized residues from the ANOVA on the vertical axis to a standard normal population on the horizontal axis. The linearity of the points on the plots suggests that the data are normally distributed. Click here for file

## References

[B1] Timar J Csuka O Remenar E Repassy G Kasler M Progression of head and neck squamous cell cancer Cancer Metastasis Rev 2005 24 1 107 127 10.1007/s10555-005-5051-5 15785876

[B2] Franceschi D Gupta R Spiro RH Shah JP Improved survival in the treatment of squamous carcinoma of the oral tongue Am J Surg 1993 166 4 360 365 10.1016/S0002-9610(05)80333-2 8214293

[B3] Ferlito A Rinaldo A Robbins KT Leemans CR Shah JP Shaha AR Andersen PE Kowalski LP Pellitteri PK Clayman GL Changing concepts in the surgical management of the cervical node metastasis Oral Oncol 2003 39 5 429 435 10.1016/S1368-8375(03)00010-1 12747966

[B4] Wu WS Wu JR Hu CT Signal cross talks for sustained MAPK activation and cell migration: the potential role of reactive oxygen species Cancer Metastasis Rev 2008 27 2 303 314 10.1007/s10555-008-9112-4 18299806

[B5] Nelson KK Melendez JA Mitochondrial redox control of matrix metalloproteinases Free Radic Biol Med 2004 37 6 768 784 10.1016/j.freeradbiomed.2004.06.008 15304253

[B6] Nelson KK Ranganathan AC Mansouri J Rodriguez AM Providence KM Rutter JL Pumiglia K Bennett JA Melendez JA Elevated sod2 activity augments matrix metalloproteinase expression: evidence for the involvement of endogenous hydrogen peroxide in regulating metastasis Clin Cancer Res 2003 9 1 424 432 12538496

[B7] Yang JQ Zhao W Duan H Robbins ME Buettner GR Oberley LW Domann FE v-Ha-RaS oncogene upregulates the 92-kDa type IV collagenase (MMP-9) gene by increasing cellular superoxide production and activating NF-kappaB Free Radic Biol Med 2001 31 4 520 529 10.1016/S0891-5849(01)00613-X 11498285

[B8] Ye H Yu T Temam S Ziober BL Wang J Schwartz JL Mao L Wong DT Zhou X Transcriptomic dissection of tongue squamous cell carcinoma BMC Genomics 2008 9 1 69. 10.1186/1471-2164-9-69 18254958 PMC2262071

[B9] O'Donnell RK Kupferman M Wei SJ Singhal S Weber R O'Malley B Cheng Y Putt M Feldman M Ziober B Gene expression signature predicts lymphatic metastasis in squamous cell carcinoma of the oral cavity Oncogene 2005 24 7 1244 1251 10.1038/sj.onc.1208285 15558013

[B10] Toruner GA Ulger C Alkan M Galante AT Rinaggio J Wilk R Tian B Soteropoulos P Hameed MR Schwalb MN Association between gene expression profile and tumor invasion in oral squamous cell carcinoma Cancer Genet Cytogenet 2004 154 1 27 35 10.1016/j.cancergencyto.2004.01.026 15381369

[B11] Ziober AF Patel KR Alawi F Gimotty P Weber RS Feldman MM Chalian AA Weinstein GS Hunt J Ziober BL Identification of a gene signature for rapid screening of oral squamous cell carcinoma Clin Cancer Res 2006 12 20 Pt 1 5960 5971 10.1158/1078-0432.CCR-06-0535 17062667

[B12] R_Development_Core_Team: R R: A language and environment for statistical computing R Foundation for Statistical Computing 2006

[B13] Gentleman RC Carey VJ Bates DM Bolstad B Dettling M Dudoit S Ellis B Gautier L Ge Y Gentry J Bioconductor: open software development for computational biology and bioinformatics Genome Biol 2004 5 10 R80. 10.1186/gb-2004-5-10-r80 15461798 PMC545600

[B14] Yu T Ye H Chen Z Ziober BL Zhou X Dimension reduction and mixed-effects model for microarray meta-analysis of cancer Front Biosci 2008 13 2714 2720 10.2741/2878 17981746

[B15] Irizarry RA Bolstad BM Collin F Cope LM Hobbs B Speed TP Summaries of Affymetrix GeneChip probe level data Nucleic Acids Res 2003 31 4 e15 10.1093/nar/gng015 12582260 PMC150247

[B16] Petruzzelli GJ The biology of tumor invasion, angiogenesis and lymph node metastasis ORL J Otorhinolaryngol Relat Spec 2000 62 4 178 185 10859518 10.1159/000027744

[B17] Ye H Wang A Lee BS Yu T Sheng S Peng T Hu S Crowe DL Zhou X Proteomic Based Identification of Manganese Superoxide Dismutase 2 (SOD2) as a Metastasis Marker for Oral Squamous Cell Carcinoma Cancer Genomics Proteomics 2008 5 2 85 94 18460737 PMC2890249

[B18] Lo WY Tsai MH Tsai Y Hua CH Tsai FJ Huang SY Tsai CH Lai CC Identification of over-expressed proteins in oral squamous cell carcinoma (OSCC) patients by clinical proteomic analysis Clin Chim Acta 2007 376 12 101 107 10.1016/j.cca.2006.06.030 16889763

[B19] Toh Y Kuninaka S Mori M Oshiro T Ikeda Y Nakashima H Baba H Kohnoe S Okamura T Sugimachi K Reduced expression of manganese superoxide dismutase mRNA may correlate with invasiveness in esophageal carcinoma Oncology 2000 59 3 223 228 10.1159/000012165 11053990

[B20] Kwee JK Mitidieri E Affonso OR Lowered superoxide dismutase in highly metastatic B16 melanoma cells Cancer Lett 1991 57 3 199 202 10.1016/0304-3835(91)90157-D 2032208

[B21] Yan T Oberley LW Zhong W St Clair DK Manganese-containing superoxide dismutase overexpression causes phenotypic reversion in SV40-transformed human lung fibroblasts Cancer Res 1996 56 12 2864 2871 8665527

[B22] Zhong W Oberley LW Oberley TD Yan T Domann FE St Clair DK Inhibition of cell growth and sensitization to oxidative damage by overexpression of manganese superoxide dismutase in rat glioma cells Cell Growth Differ 1996 7 9 1175 1186 8877099

[B23] Church SL Grant JW Ridnour LA Oberley LW Swanson PE Meltzer PS Trent JM Increased manganese superoxide dismutase expression suppresses the malignant phenotype of human melanoma cells Proc Natl Acad Sci USA 1993 90 7 3113 3117 10.1073/pnas.90.7.3113 8464931 PMC46247

[B24] Cullen JJ Weydert C Hinkhouse MM Ritchie J Domann FE Spitz D Oberley LW The role of manganese superoxide dismutase in the growth of pancreatic adenocarcinoma Cancer Res 2003 63 6 1297 1303 12649190

[B25] Ough M Lewis A Zhang Y Hinkhouse MM Ritchie JM Oberley LW Cullen JJ Inhibition of cell growth by overexpression of manganese superoxide dismutase (MnSOD) in human pancreatic carcinoma Free Radic Res 2004 38 11 1223 1233 10.1080/10715760400017376 15621700

[B26] Liu R Oberley TD Oberley LW Transfection and expression of MnSOD cDNA decreases tumor malignancy of human oral squamous carcinoma SCC-25 cells Hum Gene Ther 1997 8 5 585 595 10.1089/hum.1997.8.5-585 9095410

[B27] Izutani R Asano S Imano M Kuroda D Kato M Ohyanagi H Expression of manganese superoxide dismutase in esophageal and gastric cancers J Gastroenterol 1998 33 6 816 822 10.1007/s005350050181 9853553

[B28] Malafa M Margenthaler J Webb B Neitzel L Christophersen M MnSOD expression is increased in metastatic gastric cancer J Surg Res 2000 88 2 130 134 10.1006/jsre.1999.5773 10644478

[B29] Kim JJ Chae SW Hur GC Cho SJ Kim MK Choi J Nam SY Kim WH Yang HK Lee BL Manganese superoxide dismutase expression correlates with a poor prognosis in gastric cancer Pathobiology 2002 70 6 353 360 10.1159/000071276 12865632

[B30] Janssen AM Bosman CB Sier CF Griffioen G Kubben FJ Lamers CB van Krieken JH van de Velde CJ Verspaget HW Superoxide dismutases in relation to the overall survival of colorectal cancer patients Br J Cancer 1998 78 8 1051 1057 9792149 10.1038/bjc.1998.626 PMC2063153

[B31] Toh Y Kuninaka S Oshiro T Ikeda Y Nakashima H Baba H Kohnoe S Okamura T Mori M Sugimachi K Overexpression of manganese superoxide dismutase mRNA may correlate with aggressiveness in gastric and colorectal adenocarcinomas Int J Oncol 2000 17 1 107 112 10853026 10.3892/ijo.17.1.107

[B32] Haapasalo H Kylaniemi M Paunul N Kinnula VL Soini Y Expression of antioxidant enzymes in astrocytic brain tumors Brain Pathol 2003 13 2 155 164 12744469 10.1111/j.1750-3639.2003.tb00015.x PMC8096025

[B33] Korenaga D Yasuda M Honda M Nozoe T Inutsuka S MnSOD expression within tumor cells is closely related to mode of invasion in human gastric cancer Oncol Rep 2003 10 1 27 30 12469139

[B34] Nozoe T Honda M Inutsuka S Yasuda M Korenaga D Significance of immunohistochemical expression of manganese superoxide dismutase as a marker of malignant potential in colorectal carcinoma Oncol Rep 2003 10 1 39 43 12469142

[B35] Qi Y Chiu JF Wang L Kwong DL He QY Comparative proteomic analysis of esophageal squamous cell carcinoma Proteomics 2005 5 11 2960 2971 10.1002/pmic.200401175 15986332

[B36] Bianchi MS Bianchi NO Bolzan AD Superoxide dismutase activity and superoxide dismutase-1 gene methylation in normal and tumoral human breast tissues Cancer Genet Cytogenet 1992 59 1 26 29 10.1016/0165-4608(92)90152-X 1555188

[B37] Soini Y Vakkala M Kahlos K Paakko P Kinnula V MnSOD expression is less frequent in tumour cells of invasive breast carcinomas than in in situ carcinomas or non-neoplastic breast epithelial cells J Pathol 2001 195 2 156 162 10.1002/path.946 11592093

[B38] Baker AM Oberley LW Cohen MB Expression of antioxidant enzymes in human prostatic adenocarcinoma Prostate 1997 32 4 229 233 10.1002/(SICI)1097-0045(19970901)32:4<229::AID-PROS1>3.0.CO;2-E 9288180

[B39] Bostwick DG Alexander EE Singh R Shan A Qian J Santella RM Oberley LW Yan T Zhong W Jiang X Antioxidant enzyme expression and reactive oxygen species damage in prostatic intraepithelial neoplasia and cancer Cancer 2000 89 1 123 134 10.1002/1097-0142(20000701)89:1<123::AID-CNCR17>3.0.CO;2-9 10897009

[B40] Vairaktaris E Yapijakis C Derka S Serefoglou Z Vassiliou S Nkenke E Ragos V Vylliotis A Spyridonidou S Tsigris C Association of matrix metalloproteinase-1 (-1607 1G/2G) polymorphism with increased risk for oral squamous cell carcinoma Anticancer Res 2007 27 1A 459 464 17352267

[B41] O-charoenrat P Leksrisakul P Sangruchi S A functional polymorphism in the matrix metalloproteinase-1 gene promoter is associated with susceptibility and aggressiveness of head and neck cancer Int J Cancer 2006 118 10 2548 2553 10.1002/ijc.21644 16353148

[B42] Cao ZG Li CZ A single nucleotide polymorphism in the matrix metalloproteinase-1 promoter enhances oral squamous cell carcinoma susceptibility in a Chinese population Oral Oncol 2006 42 1 32 38 10.1016/j.oraloncology.2004.08.015 16256416

[B43] Lin SC Chung MY Huang JW Shieh TM Liu CJ Chang KW Correlation between functional genotypes in the matrix metalloproteinases-1 promoter and risk of oral squamous cell carcinomas J Oral Pathol Med 2004 33 6 323 326 15200479 10.1111/j.1600-0714.2004.00214.x

[B44] Burdon RH Superoxide and hydrogen peroxide in relation to mammalian cell proliferation Free Radic Biol Med 1995 18 4 775 794 10.1016/0891-5849(94)00198-S 7750801

[B45] Manna SK Zhang HJ Yan T Oberley LW Aggarwal BB Overexpression of manganese superoxide dismutase suppresses tumor necrosis factor-induced apoptosis and activation of nuclear transcription factor-kappaB and activated protein-1 J Biol Chem 1998 273 21 13245 13254 10.1074/jbc.273.21.13245 9582369

