# Supplementary material for: Deregulation of manganese superoxide dismutase (SOD2) expression and lymph node metastasis in tongue squamous cell carcinoma
Source: BMC Cancer. 2010 Jul 9;10:365. doi: 10.1186/1471-2407-10-365 (PMC2911422; doi:10.1186/1471-2407-10-365)
Supplement: Additional file 1 — Quantile-quantile plot (Q-Q plot) for the normality testing on the SOD2 expression values from cohort #1 and cohort #2. The normal Q-Q plots were constructed to compare standardized residues from the ANOVA on the vertical axis to a standard normal population on the horizontal axis. The linearity of the points on the plots suggests that the data are normally distributed. [file 1471-2407-10-365-S1.PPT]

## Slide 1
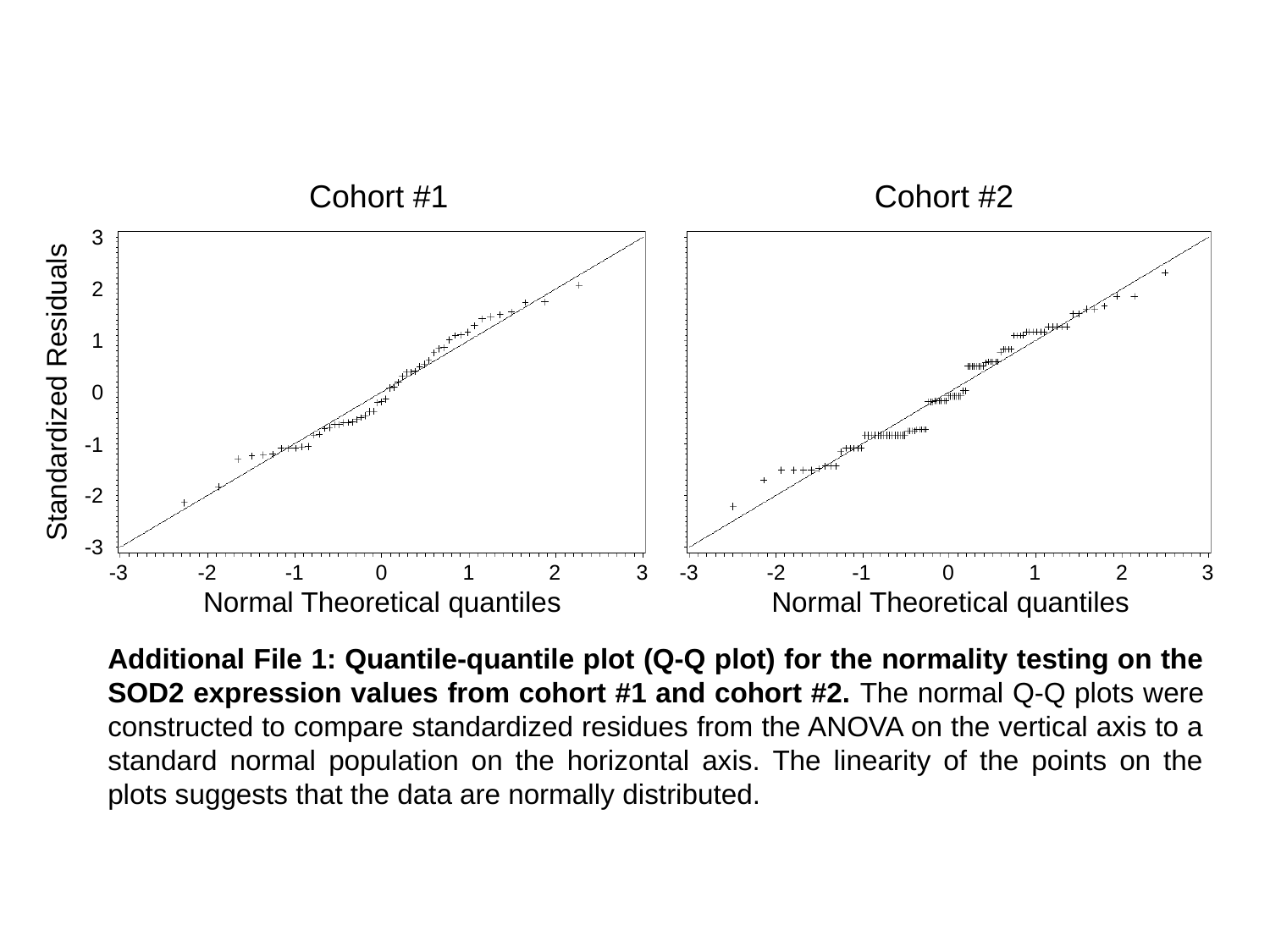

Cohort #1
Cohort #2
3
2
1
0
-1
-2
-3
-3
-2
-1
0
1
2
3
-3
-2
-1
0
1
2
3
Standardized Residuals
Normal Theoretical quantiles
Normal Theoretical quantiles
Additional File 1: Quantile-quantile plot (Q-Q plot) for the normality testing on the SOD2 expression values from cohort #1 and cohort #2. The normal Q-Q plots were constructed to compare standardized residues from the ANOVA on the vertical axis to a standard normal population on the horizontal axis. The linearity of the points on the plots suggests that the data are normally distributed.
